# Acute Myeloid Leukemia: Aging and Epigenetics

**DOI:** 10.3390/cancers12010103

**Published:** 2019-12-31

**Authors:** Polina Zjablovskaja, Maria Carolina Florian

**Affiliations:** 1 Institute of Molecular Medicine, University of Ulm, 89081 Ulm, Germany; 2 Center for Regenerative Medicine in Barcelona (CMRB), IDIBELL, Hospital Duran i Reynals, Av. Gran Via 199-203, 08908 Hospitalet de Llobregat, Barcelona, Spain; 3 Institute of Molecular Medicine, University of Ulm, James-Franck-Ring 11c, 08090 Ulm, Germany

**Keywords:** acute myeloid leukemia (AML), aging, epigenetic, hematopoietic stem cell (HSC), clonal hematopoiesis (CH), mutation

## Abstract

Acute myeloid leukemia (AML) is an aggressive hematological disorder mainly affecting people of older age. AML initiation is primarily attributed to mutations in crucial cellular regulators such as epigenetic factors, transcription factors, and signaling genes. AML’s aggressiveness and responsiveness to treatment depends on the specific cell type where leukemia first arose. Aged hematopoietic cells are often genetically and/or epigenetically altered and, therefore, present with a completely different cellular context for AML development compared to young cells. In this review, we summarize key aspects of AML development, and we focus, in particular, on the contribution of cellular aging to leukemogenesis and on current treatment options for elderly AML patients. Hematological disorders and leukemia grow exponentially with age. So far, with conventional induction therapy, many elderly patients experience a very poor overall survival rate requiring substantial social and medical costs during the relatively few remaining months of life. The global population’s age is increasing rapidly without an acceptable equal growth in therapeutic management of AML in the elderly; this is in sharp contrast to the increase in successful therapies for leukemia in younger patients. Therefore, a focus on the understanding of the biology of aging in the hematopoietic system, the development of appropriate research models, and new therapeutic approaches are urged.

## 1. Introduction

Acute myeloid leukemia (AML) is a heterogeneous hematological disease which is mainly characterized by the block of myeloid differentiation and expansion of immature myeloid progenitors (blasts) in the bone marrow (BM) of patients [1]. While statistically being a relatively rare cancer type (1.1% of all new cancer cases), according to the American Cancer Society’s [2] prediction, in the United States, AML will affect approximately 20,000 people in 2019. Acute myeloid leukemia, like many other cancers, is a disease commonly found in elderly people with the average age at diagnosis being 68 years according to NIH (National Institutes of Health) SEER (Surveillance, Epidemiology and End Results) database. Despite the existence of a long history of leukemia research, spanning more than half a century, long-term survival of elderly AML patients remains remarkably low [3]. Nevertheless, the understanding of AML biology, including molecular events leading to AML development and cellular hierarchy of the disease, has consistently improved over the last several years. Here, we review the current knowledge on AML development with a particular focus on the role of aging in leukemogenesis and disease progression.

## 2. AML Development

### 2.1. AML Initiating Events

According to the conventional view, AML develops from somatically acquired driver mutations that create a growth advantage for carrying cells [4,5,6]. Indeed, the presence or absence of specific genetic mutations is one of the main parameters for stratifying AML patients and defining the treatment protocol [7]. However, it is worth noting that driver mutations confer to the cell a fitness advantage that might be context dependent, and alterations of the microenvironment can change the relative competitiveness of a cell clone [8,9,10]. This aspect might be particularly relevant in elderly patients who may harbor mutated clones for a longer time and develop disease only when the microenvironment becomes permissive. Therefore, an immediate growth advantage might not be the only parameter to define driver mutations in elderly AML patients. This concept also extends to other cancers affecting elderly patients and could possibly explain why mutations accumulate linearly over an individual’s lifespan while cancer incidence raises exponentially with age [9].

In a study recently published by Papaemmanuil et al. [6], the presence of driver mutations in 111 pre-defined cancer genes was assessed in a cohort of 1540 patients. At least one driver mutation was identified in 96% of samples and two or more in 86% of samples. According to The Cancer Genome Atlas Research (TCGA) Network [11], genes which have mutations that are involved in the development of AML can be organized based on their biological function into nine categories: activated signaling genes (e.g., *FLT3*), DNA methylation-related genes (e.g., *DNMT3A*, *TET2*, *IDH2*), chromatin-modifying genes (e.g., *MLL-X* fusion genes, *ASXL1*, *KDM6A*, *EZH2*), gene encoding nucleophosmin (*NPM1*), myeloid transcription factor genes (e.g., *RUNX1*, *CEBPA*), transcription factor fusions (e.g., *PML-RARA*, *RUNX1-RUNX1T1*), tumor-suppressor genes (e.g., *TP53*, *WT1*), spliceosome complex genes (e.g., *U2AF1*), and cohesin complex genes (e.g., *SMC1A*, *SMC3*, *STAG2*, *RAD21*). Importantly, in most AML cases various mutations are co-occurring in different combinations, adding an additional level of complexity and heterogeneity to the disease. At the same time, certain genes or even whole gene categories are mutually exclusive, suggesting complex biological relationships among the driver mutations [11].

The very early leukemia-initiating event is still a rather controversial topic, mainly because in most cases AML arises without any detectable early symptoms, and patients usually present with the acute complications of bone marrow failure later in time [12]. The accumulation of somatic mutations in hematopoietic stem/progenitor cells happens also in individuals that eventually do not develop disease but might present with clonal hematopoiesis (termed age-related clonal hematopoiesis (ARCH)) [8,13]. According to a study by Abelson et al. [8], it is possible to discriminate ARCH from pre-AML many years before malignant transformation, and some specific mutations are linked to a higher risk of disease development (mainly *TP53* and *U2AF1*). As well, it is highly likely that cell extrinsic factors (exogenous stressors and alterations of the bone marrow microenvironment) and alterations in cell polarity [14] are major contributors to the trajectory of disease development. Recently, many reports have been dealing with the alterations in hematopoietic stem cell polarity and in the aged bone marrow microenvironment offering new insights into these topics [15,16,17].

### 2.2. Mutations in Epigenetic Modifiers

Mutations in epigenetic modifiers are found in a huge proportion of AML patients [11] and, therefore, represent an important category in the discussion about the AML-initiating events. Epigenetic alterations are defined as changes in gene function that are inheritable through cell divisions but are not caused by DNA sequence changes [18]. The epigenetic modifications that are primarily discussed in the context of AML are DNA methylation, DNA hydroxymethylation, histone acetylation, and histone lysine methylation [19].

Deoxyribonucleic acid methylation occurs as a result of a methyl group transferred to the fifth carbon in the cytosine nucleotide by DNA methyltransferases (DNMTs) (e.g., DNMT3A), which leads to the formation of 5-methylcytosin (5mC). This mostly happens in the context of a cytosine being followed by a guanine in the DNA sequence (CpG sites) [20,21]. Deoxyribonucleic acid hydroxymethylation is the result of 5mC oxidation by ten-eleven translocation (TET) enzymes (e.g., TET2) which leads to the formation of 5-hydroxymethylcytosine (hmC) [22]. Histone modifications occur predominantly at the histone N-terminal tail protruding from the nucleosomes. Histone lysine acetyltransferases (KATs) and lysine methyltransferases (KMTs; e.g., KMT2A or MLL and EZH2) are groups transferring acetyl and methyl, respectively, to lysine’s ε-amino group. The reverse process of lysine deacetylation and demethylation is governed by lysine demethylases (KDMs; e.g., LSD1) and histone deacetylases (HDACs) respectively [23].

The effect of epigenetic modifications on gene expression depends on the type of modification. For instance, DNA methylation has a mainly repressive function, since it inhibits the association between DNA-binding factors and their target DNA regions, and at the same time allows binding of specific repressive complexes [24]. Histone modifications act through affecting chromatin compaction and recruiting various effector proteins and may have both repressing and activating functions [25]. Mutations in epigenetic modifiers may result in global alterations of epigenetic and transcriptional programs and lead to the development of pre-malignant and malignant conditions (reviewed in more detail in Reference [19]).

In addition to mutations in epigenetic modifiers, changes in the epigenetic landscape can be caused by alterations in proteins involved in epigenetic regulation indirectly. For instance, isocitrate dehydrogenase (IDH) enzymes are required for conversion of isocitrate to α-ketoglutarate, that, in turn, is utilized as a co-substrate by various enzymes such as histone demethylases and TET family hydroxylases [26]. Mutant IDH1 and IDH2 catalyze the reduction of α-ketoglutarate to the structurally similar oncometabolite 2-hydroxyglutarate [27] which acts as a competitive inhibitor of α-ketoglutarate-dependent enzymes including TET family hydroxylases [26]. These mutations are associated with a hypermethylation signature, altered gene expression, and impaired hematopoietic differentiation [28,29,30]. Mutations in IDH1 or IDH2 are found in 20% of AML cases [11].

### 2.3. Pre-Leukemic State

The group of Ravindra Majeti [31,32] performed sets of experiments based on fluorescence activated cell sorting (FACS) separation of the leukemic cells from the residual non-leukemic hematopoietic stem cells (HSCs) in AML samples. Sequencing of these populations revealed that some but not all mutations present in leukemic cells were also found in the “functionally normal” residual HSCs. This suggested the existence of ancestor cells containing pre-leukemic mutations which further evolve into leukemia through gradual acquisition of additional (late) mutations [31,32]. Interestingly, in the subset of mutations discovered to be pre-leukemic, mutations in genes associated with epigenetic mechanisms (DNA methylation-related genes, chromatin-modifying genes, and cohesin complex genes) were significantly overrepresented. The same gene categories were underrepresented in the subset of late mutations. Mutations in the activated signaling genes, on the contrary, were overrepresented in the late gene subset. Based on this observation, a model was suggested, where the earliest leukemogenic events happen in “landscaping” genes involved in epigenetic regulation and are followed by mutations leading to increased activation of signaling pathways and cellular proliferation [31].

In 14% of cases, AML evolves after a myelodysplastic syndrome (MDS) [33], a hematologic disorder often described as a pre-AML condition. Myelodysplastic syndrome is characterized by abnormal BM and blood cell morphology and ineffective hematopoiesis with the frequency of blasts in the blood or BM below 20% [34]. It is considered to be evolved into full blown AML when the frequency of blasts reaches or exceeds 20% [35]. The two categories of genes most commonly mutated in MDS are splicing factors and epigenetic regulators. Interestingly, many genes found to be recurrently mutated in AML are also mutated in MDS, for instance, *DNMT3A*, *TET2*, and *ASXL1* [36]. In a recent study, Chen et al. [37] performed clonality analysis in samples obtained from patients that progressed from MDS into secondary AML. The samples were collected during both MDS and AML stages of the disease and were further fractionated into stem cells, pre-malignant stem cells, and blasts. The data revealed the existence of distinct clonal architecture in stem cell compartments with a higher number of subclones compared to blast cell compartments. In addition, in three out of seven patients, clones that were strongly expanded at the AML stage and were detectable in MDS stem cells were hardly detectable in the MDS blasts. Based on these observations, the authors suggested that secondary AML does not develop linearly from MDS stem cells through an MDS blast axis, but rather evolves in parallel with MDS through AML stem cells branching from MDS stem cells [37].

### 2.4. Leukemic Stem Cells

Over the last decades, multiple studies revealed the cellular heterogeneity of AML and the existence of a hierarchical relationship among different populations of leukemic cells [38,39,40]. In analogy to hematopoietic stem cells (HSCs) at the head of the hierarchy of the hematopoietic system, AML originates from a small population of cells which are characterized by a very high (potentially unlimited) self-renewal capacity. These cells are named leukemic stem cells or leukemia-initiating cells (LICs) due to the fact of their ability to give rise to leukemia after re-transplantation into secondary recipients. Like in the case of HSCs, the increased self-renewal capacity of LICs comes at the cost of reduced proliferation speed [41]. This property of LICs makes them a difficult target for conventional anti-cancer therapies that normally affect highly proliferative bulk leukemia cells. Leukemia-initiating cells are deemed responsible for tumor relapse which often happens with increased aggressiveness [7,42]. Therefore, the holy grail of research on leukemia is finding a way to specifically eradicate quiescent LICs.

### 2.5. Cell of Origin and “Cellular Context”

Acute myeloid leukemia is often characterized by an over-proliferation of functionally impaired immature myeloid progenitors. However, in 1997, Bonnet and Dick [38] suggested that a transforming event leading to the initiation of AML is occurring in primitive hematopoietic cells rather than in committed progenitors. This suggestion was based on immuno-phenotypical resemblance of the identified human AML LICs and normal human HSCs where both populations were CD34^+^CD38^−^ [38]. This concept was further supported by the identification of several fusion proteins associated with myeloid leukemias or their transcripts in normal HSCs and various non-myeloid cell types of AML patients, suggesting that acquisition of the mutations occurred in an immature cell capable of giving rise to cells of multiple lineages [43,44,45]. However, later viral transduction of various hematopoietic populations with fusion oncoproteins, such as MLL-AF9, MLL-ENL, MOZ-TIF2 as well as fusion oncoprotein knock-in models, revealed that progenitor cells can also be transformed [46,47,48,49,50,51]. Importantly, Krivtsov et al. [46] demonstrated that LICs isolated from progenitor-derived leukemia presented with a reactivated self-renewal-associated gene expression program. Additionally, several reports demonstrated that aggressiveness of the resulting leukemia depended on the cell of origin [47,48,49]. For example, MLL-AF9 expression in HSCs results in significantly more aggressive leukemia compared to the one arising from granulocyte-monocyte progenitor (GMP) cells. In addition, HSC-derived AML showed higher frequency of LICs and lower responsiveness to chemotherapy treatment [48]. In agreement, Siriboonpiputtana et al. [52] have demonstrated that self-renewal of LICs in HSC-derived AML is maintained through different molecular mechanisms compared to progenitor-derived AML, while phenotypically these two AMLs are indistinguishable. This suggests that genetically and phenotypically identical AMLs deriving from different cells of origin may react differently to the same treatment and emphasizes the importance of the “cellular context” for AML transformation.

## 3. Contribution of Aging to AML

One of the main risk factors for AML development is aging. However, it is not yet fully understood which aging-associated alterations contribute to leukemogenesis and to what extent. Organismal aging is accompanied by biochemical and cellular changes. Among the main hallmarks of aging are genomic and epigenomic alterations, deregulated protein homeostasis and nutrient sensing, mitochondrial disfunction, cellular senescence, and stem cell malfunction which are all considered to potentially contribute to an increased risk of disease development [53]. Multiple studies have been performed in order to characterize aging-associated alterations and explain the increased predisposition of aged individuals to cancers [54,55,56,57]. Here, we focused on genetic and epigenetic alterations of hematopoietic cells as one of the primary causative factors leading to AML development (Table 1). In addition, we mainly reviewed research data that were based on murine models and on clinical data from patients. However, we acknowledge the importance of other models used to characterize molecular mechanisms of leukemic transformation and aging.

### 3.1. Accumulation of Somatic Mutations upon Aging

One of the first mechanisms suggested to be responsible for cellular aging and cancerous transformation was the accumulation of DNA lesions during the lifespan of most individuals [73,74]. Mutations in DNA continuously arise in normal tissues as a result of spontaneous errors in biochemical processes within the cell or due to the exposure to environmental and intrinsic mutagenic factors (UV light, mutagenic chemicals, reactive oxygen species, diet, etc.). In an attempt to maintain genomic integrity, cells rely on a number of pathways which together enable a so-called DNA damage response. This mechanism represents a cascade of events including lesion recognition, signal transduction, and repair events which ultimately lead to either mutation repair or to death of the affected cell (reviewed in Reference [75]). However, errors in DNA damage response may result in survival of the mutated cell and potentially propagation of the mutation to the progeny of this cell. Because of its nature, stem cells possess the highest capacity to maintain and spread the acquired mutations through self-renewal and differentiation steps into different cellular lineages [76]. To date, multiple studies have reported a 2–3 fold increase in mutation frequency in aged cells compared to young or young adult cells for different tissues [77,78] including human hematopoietic stem cells [58]. This latter study [58] in particular showed that mutations accumulate linearly with a rate of 14 base substitutions per cell per year. It has been suggested that one of the reasons behind the accumulation of mutations in HSCs is quiescence of these cells which leads to DNA repair attenuation [59]. Further, it was shown that upon stimulation into the cell cycle, HSCs were able to repair DNA damage regardless of age [59]. This hypothesis was consistently supported by Moehrle et al. [61] who showed that both young and aged HSCs tend to leave their quiescent state upon DNA damage in vivo. Interestingly, the same study demonstrated that the ability of aged HSCs to repair DNA damage was not impaired compared to young HSCs [61].

Exome sequence comparison of the AML sample and the HSPC sample from healthy individuals revealed no significant difference in mutation number and spectrum between leukemic and normal cells within one age category. At the same time, the number of mutations in both the AML sample and the normal HSPC sample positively correlated with their age. Based on these observations, the authors suggest that the majority of mutations present in AML cells are “background” mutations and do not contribute to leukemogenesis [60]. It has also been suggested that the 2–3 fold increase in mutation frequency observed in aged cells compared to young adults’ cells is unlikely to be sufficient to explain the exponential increase in leukemia initiation observed upon aging [57]. This suggests the likely existence of additional factors contributing to leukemogenesis in aged patients.

### 3.2. Clonal Hematopoiesis in Elderly

Large-scale studies based on whole-exome sequencing of peripheral blood cells from persons unselected for cancers and hematological disorders revealed another property of the aged hematopoietic system—clonal expansion of cells carrying somatic mutations in healthy older adults, termed clonal hematopoiesis (CH) [79,80]. In more than 10% of people over 80 years old, clonal expansion of blood cells with somatic mutations was observed, whereas only 1% of people younger than 50 years presented with the same clonal composition of blood. At the same time, given the fact that detection of minor subclones depends on the technical accuracy of the sequencing methods, it is possible that improving the accuracy of next-generation sequencing in the future will reveal even higher clonality rates [81].

Clonal hematopoiesis was shown to be associated with the increased risk of development of hematologic malignancies [79,80]. Importantly, mutational analysis of genes involved in age-related CH in samples obtained from patients 6 years before AML diagnosis revealed differences in the mutational landscape and clonal expansion between pre-AML cases and the control group [8]. These data suggest that AML development through clonal evolution occurs over many years and that AML-free CH can be distinguished from pre-AML years before the disease onset.

In addition to cell-intrinsic forces driving the age-associated CH, such as acquisition of driver mutations, the role of the aged microenvironment in promoting this potentially leukemogenic clonal expansion has been suggested [82]. Indeed, some recent reports highlighted profound alterations in the BM microenvironment on aging which might play important roles in disease progression, or it might be critical to tailor the chemotherapeutic approach for the elderly [16,17,83,84]. Interestingly, the three genes most frequently found to be involved in CH in healthy aged individuals are *DNMT3A*, *TET2*, and *ASXL1*. These genes are epigenetic regulators which have previously been reported to be involved in MDS and AML development [79,80]. Based on this observation, Akunuru and Geiger [85] suggest the hypothesis of “epigenetic clonality”, where the aged hematopoietic environment supports the expansion of cells with a certain epigenetic landscape.

Importantly, mutations in epigenetic regulators *DNMT3A*, *TET2*, *IDH1/IDH2*, *ASXL1* have been shown to persist in complete remission (CR) patients [31,86]. For example, in a study by Rothenberg-Thurley et al. [87], it was reported that the persistence of these driver mutations was associated with worse prognosis, and it was most common in elderly patients. Therefore, monitoring the persistence of pre-leukemic clones after first remission was suggested to be critical to guide post-remission treatment. Indeed, allogenic transplantation abrogated the risk of relapse and, therefore, the analysis of persistent mutations during remission might provide more valuable information in terms of risk stratification than only the genetic analysis of pre-treatment samples [80,87,88]. Interestingly, minimal residual disease (MRD) measured by flow cytometry or qPCR was apparently not significantly correlated to persistent mutations, inducing to hypothesize a different mechanism for disease relapse than the survival of a small number of resistant leukemic cells.

### 3.3. Age-Associated Epigenetic Changes

In the same way as mutations accumulate throughout a person’s lifetime, epigenetic alterations were reported to increase upon aging. Analysis of DNA methylation and histone acetylation levels performed on monozygotic tweens, where age of tween-pairs was in a range between 3 and 74 years old, revealed that epigenetic difference between tweens was significantly higher in older tween-pairs than in younger ones [65]. This suggests that, over the course of a lifetime, our epigenetic landscape diverges from the original one. These random epigenetic changes were collectively termed “epigenetic drift” [65]. Moreover, large-scale methylome analysis has shown that DNA methylation patterns can reliably predict chronological age [68,89,90]. Interestingly, donor HSCs transplanted into recipient patients of different age have been shown to maintain their chronological DNA methylation age, which suggests that DNA methylation age is an intrinsic property of the cell and is not affected by the environment [91]. Further, multiple studies reported the presence of various types of epigenetic alterations as well as changes in the expression of epigenetic regulators in aged hematopoietic cells [63,64,66,67,69]. For example, a comprehensive study involving the analysis of transcriptome, DNA methylome, and histone modifications in young and aged murine HSCs described global epigenetic changes associated with stem cell aging. The authors demonstrated the reduced expression of DNA methyltransferases, the key epigenetic regulators and altered positioning of some regulatory histone marks like H3K4me3, H3K27me3, and H3K36me3 in aged cells [63]. Additionally, analysis of the epigenetic landscape of an HSC-enriched population from young (18–30 years) and aged (65–75 years) healthy donors, performed by Adelman et al. [67], revealed an age-associated reduction in H3K4me1, H3K27ac, and H3K4me3, as well as altered DNA methylation in aged cells.

Furthermore, Grigoryan et al. [70] have shown that aged murine HSCs display an increased nuclear volume and altered nuclear shape. These alterations have been shown to be associated with reduced levels of the nuclear envelope protein Lamin A/C in aged HSCs. In addition, the authors reported an altered distribution of a H3K9me2 heterochromatin mark in aged HSCs. Interestingly, treatment with CASIN (a Cdc42-activity inhibitor) [71,92,93] restored Lamin A/C levels, H3K9me2 peripheral localization, and nuclear volume in aged HSCs to the levels observed in young HSCs. Alterations of another heterochromatin mark, H3K9me3, have been reported in aged HSCs by Djeghloul et al. [62]. Reduction in the global level of H3K9me3 in aged murine and human HSCs has been shown to be associated with reduced expression of one of the principal enzymes involved in heterochromatin formation—the methyltransferase SUV39H1 [62]. Further evidence of an altered heterochromatin structure in aged cells is provided by research performed on non-mammalian and non-hematopoietic models [94,95]. Altogether, these data suggest the presence of global changes in the nuclear structure in HSCs upon aging which, in turn, might be linked to changes in chromatin organization and gene expression [96].

Given the fact that an altered epigenetic state might result in an altered transcriptional profile and, consequently, impaired function of the cell [97], it is reasonable to hypothesize that changes in the epigenetic landscape and nuclear structure of aged hematopoietic cells, especially HSCs, might contribute to differences in the intracellular context between young and aged LICs and, therefore, result in different behaviors of “aged” AML cells compared to “young” cells despite the same genetic alterations.

In addition to genetic and epigenetic alterations, changes in the expression of multiple cellular regulators [63,72], increased expression of repetitive elements [98,99], and altered expression of different protein isoforms have been reported in aged cells [63]. However, we did not discuss these aspects in the current review.

### 3.4. Epigenetic Polarity in Aged HSC

Another type of aging-associated epigenetic alteration that has recently been reported, but not yet fully understood, is the loss of H4K16ac polarity in aged HSCs associated with increased activity of the small RhoGTPase Cdc42. Importantly, inhibition of Cdc42 activity with a small molecule compound, CASIN, which leads to restoration of H4K16ac polarity, has been shown to correlate with functional rejuvenation of HSC [71]. The same group has shown that loss of epigenetic polarity in HSCs is associated with an increased rate of symmetric self-renewing divisions in aged stem cells [93] which is in line with the phenotypic expansion of HSCs observed upon aging [100,101]. The importance of this observation is emphasized by the role of aberrant self-renewal in AML development [38]. Further research is needed to understand whether changes in epigenetic polarity contribute to leukemogenesis and whether reversal of these changes could be used as an approach to improve disease outcome.

## 4. Aging and AML

**Figure 1:**
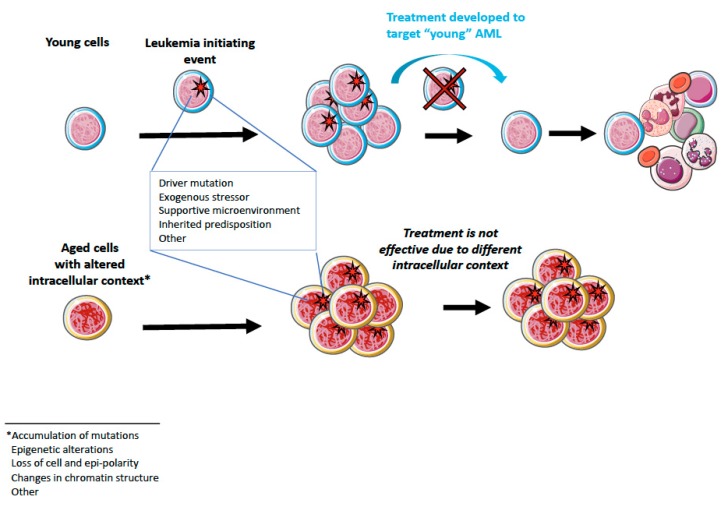
Cartoon scheme illustrating the effect of young and aged intracellular contexts on leukemic transformation. A leukemia-initiating event is indicated with a red star. Healthy hematopoietic stem and progenitor cells (HSPCs) (without red star) acquire a leukemia-initiating event which leads to expansion of the leukemic cells (with the red star). After diagnosis, young and aged leukemic cells are treated with the compound developed to target young leukemic cells. Young leukemic cells respond to the treatment and dye. Residual healthy HSPCs support the regeneration of the hematopoietic system. Aged leukemic cells do not respond to the same treatment due to the different intracellular genetic and/or epigenetic context and leukemia progression is not arrested. Figure created by using the resources provided by Servier Medical ART which is licensed under a Creative Commons Attribution 3.0 Unported License.

Discoveries over the past few years have revealed that combinations of several factors, such as co-occurrence of mutations [6], mutation order [102], cell of origin/intracellular context [48], can affect AML kinetics, development, and treatment responsiveness. Based on these observations, it became particularly evident that AML developed on the basis of aged hematopoietic cells might have distinct properties compared to “young” AML, despite similar clinical manifestation (Figure 1 and Table 2). Indeed, a study by Silva et al. [54], who performed an analysis of samples from elderly (65 to 90 years old) patients and data from the TCGA network [11], and Metzeler’s group [103] revealed that AML presents with distinct genetic and epigenetic patterns in the elderly. The authors showed that elderly patients more often accumulate mutations in epigenetic regulators than young patients. Specifically, mutations in *DNMT3A*, *TET2*, SRSF2, and *ASXL1* were found to be more frequent in elderly patients. Further, increased frequency of genetic alterations in spliceosome-related genes and genes involved in DNA repair as well as differentially methylated DNA regions have been reported in elderly AML patients [54].

The hypothesis suggesting that aging is contributing to leukemogenesis is also supported by a publication by Adelman et al. [67]. Analyses comparing age-associated epigenetic changes in HSC-enriched population and AML-associated epigenetic alterations (differentially methylated regions and enrichment/depletion of several histone marks) revealed similarities between age-associated and AML-associated epigenetic alterations [67]. Moreover, the role of the epigenetic drift in MDS/AML development was emphasized by Maegawa et al. [66]. Using murine transgenic AML models, progressive hypermethylation of preselected genes has been shown from young to old normal BM, further to MDS, and finally to AML [66]. Interestingly, Mizukawa et al. [104] have demonstrated that genetic depletion of CDC42 in both murine and human MLL-AF9-induced AML results in a reduced rate of self-renewing divisions and blocked leukemia development [104]. Since CDC42 activity is increased upon aging in murine and human cells and is associated with impaired HSC function [71,105], the requirement of CDC42 for leukemogenesis suggests that increased activation of CDC42 in aged HSCs might make them more vulnerable to leukemic transformation. In addition, CH, often observed in aged people [79,80], might underlie the differences between young- and aged-patient AML. However, whether the presence of clonal hematopoiesis is associated with worse outcome is still quite controversial and has not been yet clarified [87,106,107,108,109].

Another aging-related feature that has been associated to increased incidence of MDS is telomere dysfunction [110]. Employing an inducible telomerase reverse transcriptase-estrogen receptor (TERT^ER^) mouse model, it was demonstrated that persistent physiological DNA damage (from eroded telomeres) drives MDS in mice by inducing aberrant RNA splicing [110]. In human, short telomeres syndromes are also associated with accelerated aging syndromes characterized by diverse clinical manifestations and with bone marrow failure and idiopathic pulmonary fibrosis being frequent manifestations [111,112]. Similarly, skewing in X-chromosome inactivation has been more frequently detected in blood from elderly women [113] and deregulation of X-chromosome inactivation in the hematopoietic compartment is known to cause typical aging-like myelodysplastic syndromes (MDS) with 100% penetrance in mice [114].

## 5. Therapy

### 5.1. Currently Available Treatment Options

The standard approach to treat AML patients is chemotherapy which in some cases might be followed by stem cell transplantation. Conventional chemotherapy, commonly referred to as “7 + 3”, consists of 7 days cytarabine and 3 days anthracycline (daunorubicin) treatment [115]. These drugs act by inhibiting DNA and RNA synthesis and, therefore, highly proliferative cells are supposed to be particularly sensitive to this treatment, including AML cells.

However, therapeutic approaches may vary depending on various factors such as patient’s age, general physical fitness, type and genetics of AML at diagnosis. For instance, patients with *FLT3* mutation, in addition to standard chemotherapy treatment, might be considered to receive the tyrosine kinase inhibitors midostaurin in the case of newly diagnosed AML [116], or gilteritinib in the case of FLT-3^+^ relapsed/refractory AML [117]. These compounds are inhibiting cells expressing wild-type as well as mutant forms of FLT3 [118,119]. Newly diagnosed AML patients with mutation in *IDH1* can be treated with the small-molecule inhibitor ivosidenib [120], and adult patients with relapsed/refractory AML with *IDH2* mutation with enasidenib [121]. Ivosidenib and enasidenib target mutant forms of the IDH1/2 molecules and promote differentiation of myeloid blasts thereby reducing tumor burden and aggressiveness [122,123]. In addition, a targeted therapy with gemtuzumab ozogamicin, an anti-CD33 antibody conjugated with a cytotoxic drug, was approved for treatment of CD33-positive AML [124].

Unfortunately, older and unfit patients might not be able to tolerate intensive chemotherapy; therefore, other treatment procedures are required. However, treatment alternatives for these patients are quite poor. Until recently, less intensive chemotherapy, treatment with hypomethylating agents (HMA), and best supportive care were the options suggested for older or unfit AML patients [7]. The hypomethylating agents currently in use include azacytidine and decitabine which are pyrimidine analogs that function as DNA methyltransferase inhibitors. These drugs are believed to revert DNA hypermethylation in AML cells and, thus, restore expression of critical onco-suppressor genes [19].

In 2018, the Food and Drug Administration (FDA) approved two additional drugs for patients who are 75 years or older. First, venetoclax [125]—an inhibitor of the anti-apoptotic protein BCL-2 which plays an important role in maintenance of leukemic blasts [126,127]. Venetoclax is suggested to be used in combination with HMA (azacitidine or decitabine) or low-dose cytarabine [128]. A second possible drug is glasdegib, approved in combination with low-dose cytarabine [129,130,131]. Glasdegib is a small-molecule inhibitor of the Hedgehog pathway, shown to be involved in the development of drug resistance [132].

### 5.2. Possibilities for Aging-Targeted Therapies?

Assuming the contribution of aging to leukemic development, it is tempting to speculate that an aging-targeting therapy might be a possible approach to refine or improve treatment options for AML in the elderly. Recently, several compounds with a potential to rejuvenate the hematopoietic system have been described [85,133,134]. For example, besides the aforementioned CDC42 activity inhibitor (CASIN), the senolytic compound ABT263 effectively kills senescent cells in the BM of aged mice, leading to reversal of several age-associated hematopoietic alterations [135]. Another compound that has been shown to rejuvenate aged HSCs in vivo is rapamycin, which acts through inhibition of the mTOR pathway (upregulated in aged HSCs) [136]. Other aging-associated HSC alterations suggested to be promising targets for the hematopoietic system rejuvenation therapy are, for example, autophagy [137] and reduced expression of SIRT3 and SIRT7, proteins involved in the regulation of mitochondrial function and cellular metabolism [138,139].

## 6. Perspectives

More than 30% of AML patients are 75 years or older according to NIH SEER database. At the same time, AML in the elderly (>60 years) is characterized by a lower overall survival rate and shorter remission duration compared to AML in younger adults [140]. Given these facts, the number of available treatment opportunities for elderly patients is unsatisfactory. This has also been acknowledged by international expert panels who have published recommendations for diagnosis and management of AML in adults on behalf of the European LeukemiaNet (ELN) [7].

The low number of treatment options might partially be a result of the “young-cell-focused” research. Given the obvious role of aging in leukemogenesis, research employing aged models might generate data with higher biological relevance for elderly patients. Genetically engineered models of accelerated aging with shorter lifespan as well as models with longer life spans are available [141]. However, these models might not properly reflect processes happening during physiological aging [142]. Preclinical AML studies employing chronological aging models might produce more accurate and biologically relevant data. For example, multiple murine models for various AML types are currently available for research [143]. Testing these models on the background of aged hematopoietic cells might significantly contribute to the development of the field and directly demonstrate the role of the aged environment in leukemogenesis. However, we acknowledge that making the use of chronological aging models is expensive and cumbersome. Importantly, on a clinical level, to facilitate the development of elderly AML-specific drugs, an increased enrolment of aged AML patients into clinical trials has been suggested [7].

## 7. Conclusions

AML grows exponentially with age. So far, with conventional induction therapy, many elderly patients experience a very poor overall survival rate requiring substantial social and medical costs during the relatively few remaining months of life. The global population’s age is increasing rapidly without an acceptable equal growth in therapeutic management of AML in the elderly; this is in sharp contrast to the increase in successful therapies for leukemia in younger patients. A focus on the understanding of epigenetic features and on the genome-epigenome interactions that dynamically change during the age-related transition from normal HSCs, through HSCs driving clonal hematopoiesis, to leukemic stem cells in AML will help to develop new therapeutic approaches and unravel the age-related factors inherent in AML of the elderly which mediate the inferior clinical outcome even in the presence of genetic features associated with low risk.

## Figures and Tables

**Table 1:** Aging-associated alterations in hematopoietic cells defining changes in the intra-cellular context.

Category	Type of Alteration	References
Mutations	2–3 fold increase in mutation load	[58,59,60,61]
Protein expression	Altered expression of epigenetic regulators	[62,63,64]
Epigenetic drift	DNA methylation alteration (redistribution, level change)	[65,66,67,68,69]
	Changes in histone modifications	[63,67]
Nuclear/chromatin structure alterations	Reduced lamin A/C level, changed nuclear size and shape, global changes in heterochromatin mark deposition	[62,70]
Epipolarity	CDC42 activity, H4K16ac polarity	[71]
Others (not discussed in the review)	Increased expression of repetitive elements	[63]
	Alternative protein isoforms	[63]
	Altered expression of non-epigenetic cellular regulators	[63,72]

**Table 2:** Epigenetic genes mutated in acute myeloid leukemia (AML): type of mutations and frequencies in adult and elderly patients and clinical features. (n.d.: not defined).

Gene	Type pf Mutation and Mechanism	Frequency in Adult AML (<70 y)	Frequency in Elderly AML (>75 y)	Clinical Features	Reference
*TET2*	Missense, nonsense, and frame shift mutations which cause a loss-of-function phenotype and the impairment of the catalytic activity, resulting in low levels of 5-hmc in genomic DNA	8–12%	42%	Debated prognostic value (associated with poor prognosis in AML patients with intermediate-risk cytogenetic but also reported with no clinical impact in other studies); mutually exclusive with IDH1/2 mutation	[6,7,11,103]
*DNMT3A*	Missense, nonsense, and frame shift heterozygote mutations which cause a dominant negative loss of function	19–26%	35%	Frequently occurring in AML with a normal karyotype; unfavorable prognosis; the loss of methylase activity results in hypomethylation and uncontrolled expression of multiple *HOX* genes	[11,103]
*ASXL1*	Missense, nonsense, and frame shift loss-of-function mutations; ASXL1 interacts with PCR2 and mutations decrease recruitment of PRC2 to its targets	11%	21%	Early mutation which tends to be associated with an aggressive disease and a poor overall survival	[103]
*IDH2/IDH1*	Heterozygous missense mutations which result in reduced production of α-KG and in a neomorphic gain-of-function effect, catalyzing the conversion of α-KG to 2-HG resulting in inhibition of TET2.	12–20%	15%	Hypermethylation signature, altered gene expression, and impaired hematopoietic differentiation	[11,28,103]
*STAG2, RAD21, SMC3, SMC1A*	Frameshift and missense mutations which disrupt cohesin complex assembly; these mutations act at least partially as dominant negatives	9–15%	n.d.	Mutually exclusive with unfavorable-risk cytogenetics as well as complex chromosomal changes; independent favorable risk factors in AML but associated with a shorter survival in MDS	[11,103]
*EZH2*	Missense, nonsense, and frame shift, loss-of-function mutations: EZH2 is a histone H3K27 methyl-transferase and part of PRC2. Loss-of-function mutations occur in the catalytic SET domain	1–2%	n.d.	*EZH2* inactivating mutations are associated to induction of HOXA9 expression	[11,103]
